# Studies of Performance of Cs_2_TiI_6−X_Br_X_ (Where x = 0 to 6)-Based Mixed Halide Perovskite Solar Cell with CdS Electron Transport Layer

**DOI:** 10.3390/mi14020447

**Published:** 2023-02-14

**Authors:** Kunal Chakraborty, Nageswara Rao Medikondu, Kumutha Duraisamy, Naglaa F. Soliman, Walid El-Shafai, Sunil Lavadiya, Samrat Paul, Sudipta Das

**Affiliations:** 1 Advanced Materials Research and Energy Application Laboratory, Department of Energy Engineering, North-Eastern Hill University, Shillong 793022, India; 2 Department of Mechanical Engineering, Koneru Lakshmaiah Education Foundation, Vaddeswaram 522302, India; 3 Department of Biomedical Engineering, KarpagaVinayaga College of Engineering and Technology, Chengalpattu 603308, India; 4 Department of Information Technology, College of Computer and Information Sciences, Princess Nourah Bint Abdulrahman University, P.O. Box 84428, Riyadh 11671, Saudi Arabia; 5 Security Engineering Lab, Computer Science Department, Prince Sultan University, Riyadh 11586, Saudi Arabia; 6 Department of Electronics and Electrical Communications Engineering, Faculty of Electronic Engineering, Menoufia University, Menouf 32952, Egypt; 7 Department of Information and Communication Technology, Marwadi University, Rajkot 360003, India; 8 Department of Electronics & Communication Engineering, IMPS College of Engineering and Technology, Malda 732103, India

**Keywords:** perovskite, solar cell, diffusion, efficiency

## Abstract

The present research work represents the numerical study of the device performance of a lead-free Cs_2_TiI_6−X_Br_X_-based mixed halide perovskite solar cell (PSC), where x = 1 to 5. The open circuit voltage (V_OC_) and short circuit current (J_SC_) in a generic TCO/electron transport layer (ETL)/absorbing layer/hole transfer layer (HTL) structure are the key parameters for analyzing the device performance. The entire simulation was conducted by a SCAPS-1D (solar cell capacitance simulator- one dimensional) simulator. An alternative FTO/CdS/Cs_2_TiI_6−X_Br_X_/CuSCN/Ag solar cell architecture has been used and resulted in an optimized absorbing layer thickness at 0.5 µm thickness for the Cs_2_TiBr_6_, Cs_2_TiI_1_Br_5_, Cs_2_TiI_2_Br_4_, Cs_2_TiI_3_Br_3_ and Cs_2_TiI_4_Br_2_ absorbing materials and at 1.0 µm and 0.4 µm thickness for the Cs_2_TiI_5_Br_1_ and Cs_2_TiI_6_ absorbing materials. The device temperature was optimized at 40 °C for the Cs_2_TiBr_6_, Cs_2_TiI_1_Br_5_ and Cs_2_TiI_2_Br_4_ absorbing layers and at 20 °C for the Cs_2_TiI_3_Br_3_, Cs_2_TiI_4_Br_2_, Cs_2_TiI_5_Br_1_ and Cs_2_TiI_6_ absorbing layers. The defect density was optimized at 10^10^ (cm^−3^) for all the active layers.

## 1. Introduction

A solar cell is made for the conversion of solar energy into electrical energy directly, which has undergone continuous development from the past few years due to its superior photovoltaic (PV) properties. Initially, 1st generation wafer-based PV technology was developed but the production cost was relatively higher with lower conversion ability. The production cost became lower after the introduction of thin film-based 2nd generation solar cells, but still the power conversion efficiency (PCE) remained low. However, the problems including cost and PCE were nullified after the development of thin film-based 3rd generation PV technology. Perovskite solar cells (PSCs) have a great capability towards photovoltaic applications and are rapidly emerging as 3rd generation solar cells [1,2]. The journey started with Kojima et al. 2009, with halide perovskite ABM_3_ (A: organic CH_3_NH_3_^+^; B: Pb, M: Br, I), and they recorded 3.8% of power conversion efficiency (PCE, %) [1]. Later, Yang et al. 2015, and Yin et al. 2015 further carried out extensive research on material, deposition process, fabrication methods and device structure that enhanced the PCE up to 20.1% experimentally, and theoretically 31.4% [3,4]. Hosseini et al. 2022 showed the effect of non-ideal conditions on lead-based CH_3_NH_3_PbI_3_ perovskite material [5]. Despite the higher conversion efficiency in the laboratory, it has several issues in terms of commercialization such as environment protection from the toxic lead (Pb) component, and stability under environment conditions due to the organic component used in the ABM_3_ structure. To address such issues, researchers considered using lead-free inorganic material as the absorbing material for the photovoltaic applications. Titanium (Ti)-based PSCs were introduced by Ju et al. 2018 with 1.0–1.8 eV tuneable band gap, and Chen et al. (2018) achieved 1.02 V open circuit voltage (V_OC_, V), 5.69 mA/cm^2^ current density (J_SC_, mA/cm^2^) and 56.4% fill factor (FF, %) with Cs_2_TiBr_6_ absorbing material [6,7]. Shyma et al. 2022 employed SCAPS-1D-based simulation on Sn-based perovskite material CH_3_NH_3_SnI_3_ to investigate the various parameters of the active materials and the selection of the appropriate ETL (electron transport layer) for the device [8]. On the other side, Omarova et al. 2022 showed the selection of the optimal HTL (hole transport layer), and also determined that electrodes can minimize the effects of material defects to improve the device performance [9].

By taking the knowledge from the above discussion, our research article has pinpointed several vital contributions including developing a theoretical model of a lead-free mixed halide FTO/CdS/Cs_2_TiI_6−X_Br_X_ /CuSCN/Ag-based structure where all the materials used for the SCAPS simulation are inorganic in nature, and then important physical, opto-electronic parameters such as thickness of material, device temperature and defect density of the material are optimized. For this optimization, we have analyzed the device performance parameter PCE with the variation in thickness of the active materials, followed by the device performance with the device temperature and defect density.

## 2. Device Architecture and Simulation

**Figure 1:**
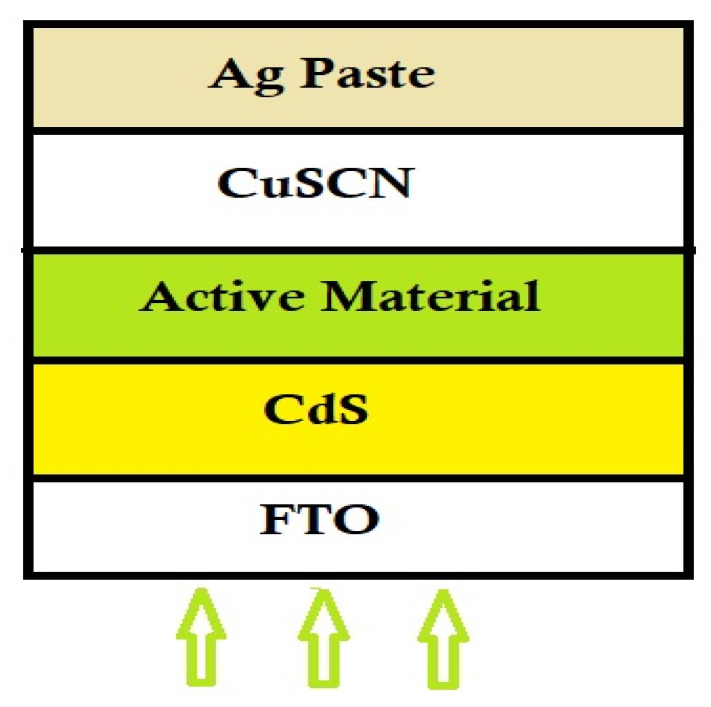
Schematic view of the proposed solar cell structure.

In our proposed FTO/CdS/Cs_2_TiI_6−X_Br_X_/CuSCN/Ag-based planar solar cell device model, the band gap of ETL (electron transport layer) materials, CdS and HTL material CuSCN are taken to be 2.4 eV, 3.26 eV and 3.4 eV, respectively, and the absorbing layer is tuneable under 1.0–1.78 eV [10,11,12,13,14,15]. The working temperature for the simulation is maintained at 27 °C with −0.8 V to 0.8 V bias voltage in the SCAPS-1D simulator. Here, Figure 1 shows the schematic view of the proposed structure. the simulation is carried out with illumination of AM 1.5 with the light power of 1000 W/m^2^ under Gaussian energy distribution, and its characteristic energy is set to 0.1 eV. With Br doping the lattice parameter, band structure and the optical properties of Cs_2_TiI_6_ can be changed. Thus, due to the doping effect, both Cs_2_TiI_2_Br_4_ and Cs_2_TiI_3_Br_3_ are suitable for solar cell applications. On the other side, based on the superior optical coincidence index and better absorption coefficient, Cs_2_TiI_2_Br_4_ and Cs_2_TiIBr_5_ are ideal for light harvesting applications [16]. The details of the device materials’ properties and active materials’ basic parameters taken for the work are shown in Table 1 and Table 2, respectively. The symbols, i.e., E_g_, denote the band gap energy; ꭓ is the electron affinity; ℇ_r_ is the relative permittivity; N_A_ is the acceptor density; N_D_ is the donor density; N_t_ is the total defect density; N_C_ is the conduction band effective density of states; N_V_ is the valence band effective density of states, respectively. The default values of some parameters and settings are as follows: electron mobility is 4.4 cm^2^/V_s_, hole mobility is 2.5 cm^2^/V_s_ and electron and hole thermal velocity is 10^7^ cm/s [17]. The Shockley–Queisser limit of a perfect photovoltaic absorbing material is around 1.3 eV [16]. Our Cs_2_TiI_6−X_Br_X_-based active material has a band gap in the range of 1.07 to 1.78 eV which can be seen in Table 1.

## 3. Results and Discussion

### 3.1. Optimization of Absorbing Layer Thickness with CdS Layer

**Figure 2:**
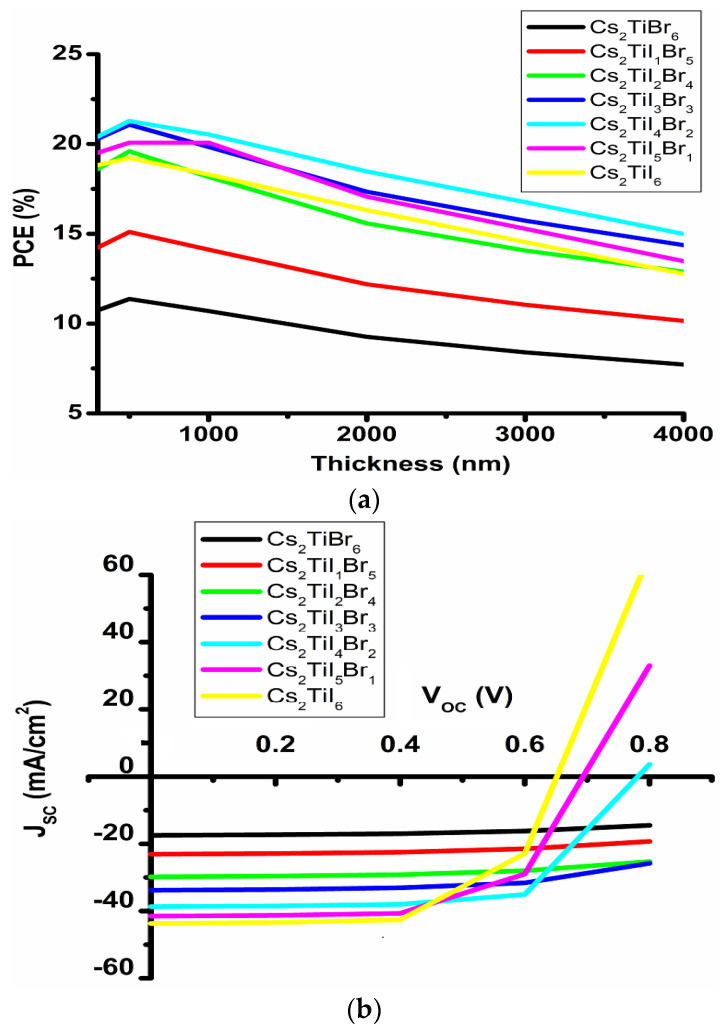

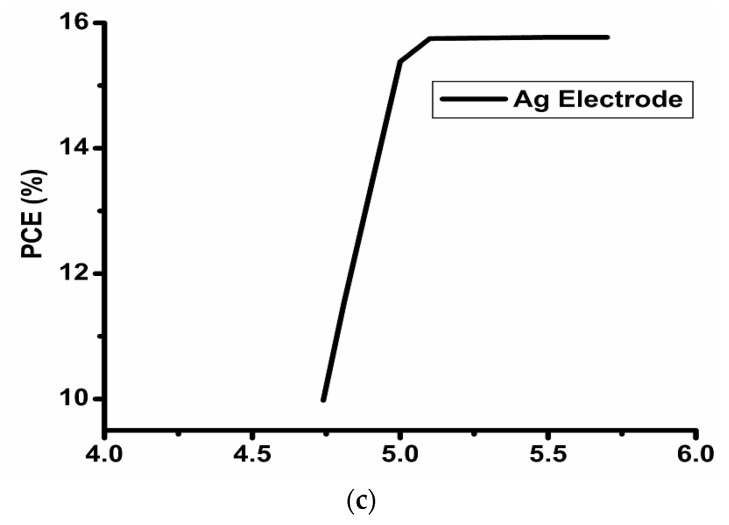
Performance of Cs_2_Ti–_6−X_Br_X_ absorbing layer with thickness variation for FTO/CdS/Cs_2_Ti–_6−X_Br_X_/CuSCN/Ag structure: (**a**) PCE, (**b**) J-V plot and (**c**) electrical properties of metal work function.

In this section, the optimization of thickness of different perovskite absorbing layers such as Cs_2_TiBr_6_, Cs_2_TiI_1_Br_5_, Cs_2_TiI_2_Br_4_, Cs_2_TiI_3_Br_3_, Cs_2_TiI_4_Br_2_, Cs_2_TiI_5_Br_1_ and Cs_2_TiI_6_ has been studied at 27 °C temperature with standard defect density (~1014 cm^−3^) and a constant series resistance [18] by varying the thickness from 0.3 to 3.0 µm. Here, Figure 2a–c represent the PCE; a J-V graph with the thickness variation as 0.3 µm, 0.4 µm, 0.5 µm, 1.0 µm, 1.5 µm, 2.0 µm, 2.5 µm, 3.0 µm, 3.5 µm and 4.0 µm for the Cs_2_Ti–_6−X_Br_X_ perovskite solar cell; and PCE with the back contacts’ metal work function. Generally, the lower thickness of absorbing material leads to a low absorption of sunlight, which has a direct impact on PCE. From Figure 2a,b, it is observed that after 0.5 µm thickness for the Cs_2_TiBr_6_, Cs_2_TiI_1_Br_5_, Cs_2_TiI_2_Br_4_, Cs_2_TiI_3_Br_3_ and Cs_2_TiI_4_Br_2_, and after 1.0 µm thickness for the Cs_2_TiI_5_Br_1_ and 0.4 µm thickness for the Cs_2_TiI_6_ absorbing layer, the PCE of the device cell starts to fall gradually. The reason behind this fall is due to the increment in thickness of the absorbing layer as the light absorption rate becomes much higher. Such excess light absorption leads to a temperature rise in the device, and thus V_OC_ starts to fall drastically due to this temperature rise [12]. With the drop in V_OC_, the PCE of the device starts to decrease. We all are aware that the PCE is a key parameter to evaluate the total annual power generation of a perovskite solar cell device-based PV (photovoltaic) module. Thus, reducing the PCE will decrease the maximum generated power (P_MAX_) in the device. Despite the V_OC_ starting to fall with thickness, the generation rate of the charge carrier increases, which has a direct impact on the enhancement of J_SC_ [19]. Such an enhancement in J_SC_ will increase the Shockley–Read–Hall (SRH) recombination [20,21]. This phenomenon could establish a V_OC_–J_SC_ relationship in the PV module [20,21,22].

(1) $$V_{\mathrm{OC}}\;=\frac{\mathrm{nKT}}{q}\ln\;(\frac{{\;J}_{\mathrm{SC}\;}}{I_{0}}\;+\;1)$$

where T = device temperature, I_0_ = reverse saturation current, q = electronic charge, n = ideality factor and K = Boltzmann constant.

From Equation (1), it is shown that the V_OC_ starts to fall as the reverse saturation current (I_0_) increases in the device. Therefore, by analyzing all the parameters we may conclude that the Cs_2_TiBr_6_, Cs_2_TiI_1_Br_5_, Cs_2_TiI_2_Br_4_, Cs_2_TiI_3_Br_3_ and Cs_2_TiI_4_Br_2_ materials are optimized at 0.5 µm; the Cs_2_TiI_5_Br_1_ material is optimized at 1.0 µm; and the Cs_2_TiI_6_ material is optimized at 0.4 µm.

From Figure 2c it is observed that the PCE increases with the higher metal work function of the Ag paste up to a certain work function value (5 eV). The reason behind such an increment in the work function value is the decrement of the height of the carrier barrier, and as a result, the metal contact becomes ohmic in nature [8]. Thus, open circuit voltages also increase.

**Figure 3:**
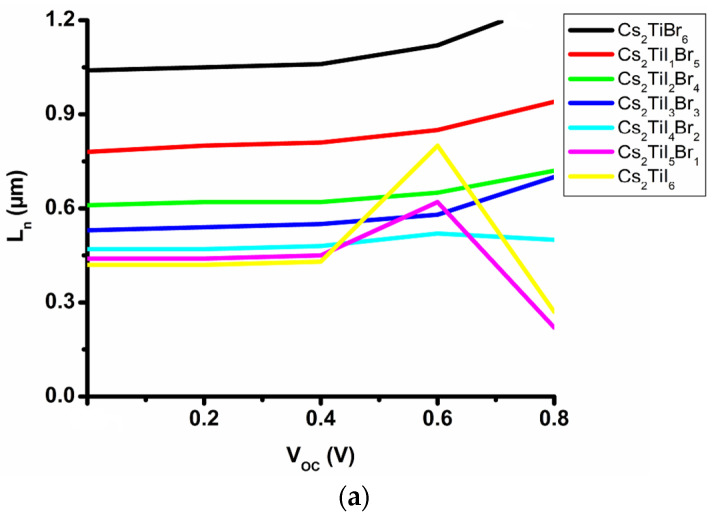

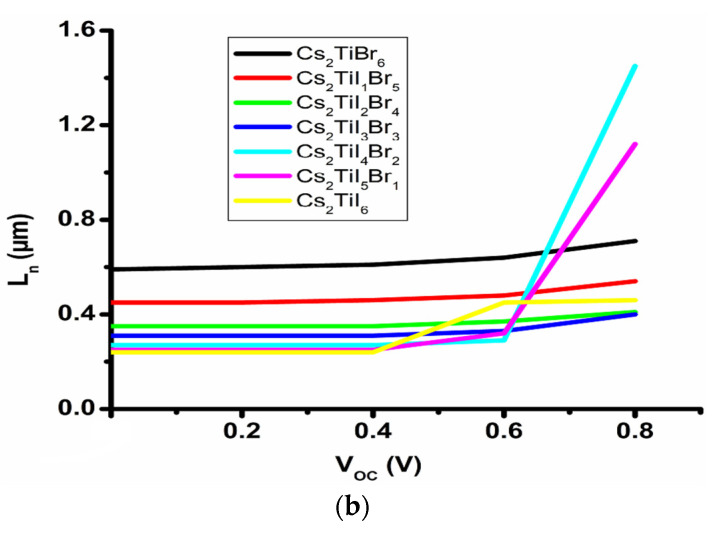
Changes in diffusion length with the open circuit voltage: (**a**) electron, (**b**) hole.

On the other hand, Figure 3a,b indicate the changes in diffusion length (L_n_) with the voltage variation at the ambient temperature (300 K). As per the observations, the diffusion length increases with the open circuit voltage (V_OC_) for all the seven absorbing materials except for Cs_2_TiI_5_Br_1_ and Cs_2_TiI_6_, where the diffusion length decreases after 0.6 V. The concentrations of electrons and holes are enhanced with the voltage which leads to an increment in diffusion length. The diffusion length of the electrons and holes should be higher than the absorbing layer thickness [8], since Cs_2_TiBr_6_, Cs_2_TiI_1_Br_5_ and Cs_2_TiI_6_ absorbing materials have a band gap above 1.50 eV which requires large energy to excite an electron in the conduction band. Thus, such materials operate in a higher temperature which can burn the device after a certain temperature. Therefore, despite the better power absorption and power generation capability of Cs_2_TiBr_6_, Cs_2_TiI_1_Br_5_ and Cs_2_TiI_6_ absorbing material, they are not suitable for PV application.

### 3.2. Optimization of Device Temperature with CdS Layer

In our study, we have varied the temperature from −10 °C (263 K) to 100 °C (373 K) at an optimized thickness (Cs_2_TiBr_6_ = 0.5 µm, Cs_2_TiI_1_Br_5_ = 0.5 µm, Cs_2_TiI_2_Br_4_ = 0.5 µm, Cs_2_TiI_3_Br_3_ = 0.5 µm, Cs_2_TiI_4_Br_2_ = 0.5 µm, Cs_2_TiI_5_Br_1_ = 1.0 µm and Cs_2_TiI_6_ = 0.4 µm) for the FTO/CdS/Cs_2_Ti–_6−X_Br_X_ /CuSCN device structure. Here, Equation (1) can be rewritten as follows [23,24]:

(2) $$V=\frac{K_{B}T}{q}\;\log\;(\frac{r^{\mathrm{if}}}{r_{0}^{\mathrm{if}}})$$

where V = V_OC_, $r^{\mathrm{if}}=I_{\mathrm{SC},\;}r_{0}^{\mathrm{if}}={\;I}_{0,\;}$n is the ideality factor, I_SC_ is the short circuit current and I_0_ is the reverse saturation current.

**Figure 4:**
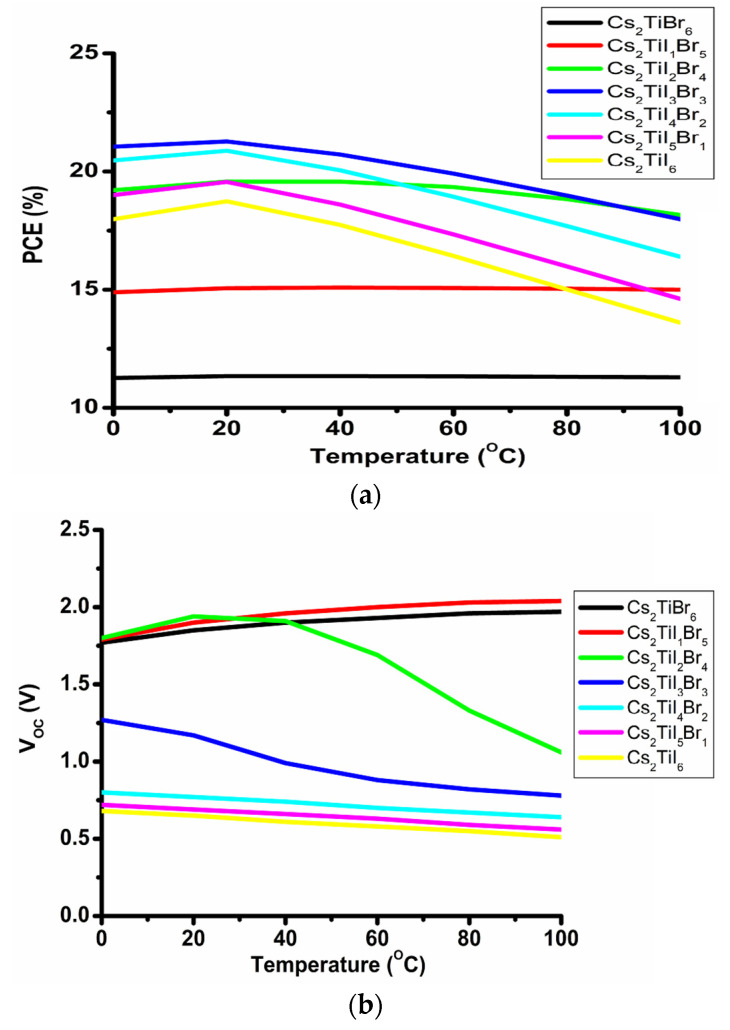
Device performance of Cs_2_Ti–_6−X_Br_X_ absorbing layer for the CdS ETL with temperature variation at optimized thickness: (**a**) PCE, (**b**) V_OC_.

From Equation (2) we can conclude that as the temperature starts to increase, the open circuit voltage (V_OC_) will decrease accordingly. The prime reason behind such a decrement in V_OC_ is the exponential inverse increment in r_0_^if^ in Equation (2), which leads to a similar exponential inverse increment in I_0_ due to the temperature rise [12]. On the other hand, the temperature increment may cause the increment in the recombination process which will lead to the increment in the short circuit current (I_SC_) [21]. Figure 4a,b show the changes in the PCE and V_OC_ with the device temperature for the absorbing materials and suggest that up to 40 °C, the V_OC_ increases quite significantly for the Cs_2_TiBr_6_, Cs_2_TiI_1_Br_5_ and Cs_2_TiI_2_Br_4_ absorbing material, and after that temperature there is a significant change observed in the V_OC_. Such a higher V_OC_ starts to increase the P_MAX_. The reasons behind such peculiarities are the higher band gap of the Cs_2_TiBr_6_ (1.78 eV), Cs_2_TiI_1_Br_5_ (1.58 eV) and Cs_2_TiI_2_Br_4_ (1.38 eV) perovskite layers, and as the temperature starts to increase initially, the band gap of the materials starts to reduce, leading to a higher increment in the V_OC_ for the Cs_2_TiBr_6_ (1.78 eV) and Cs_2_TiI_1_Br_5_ (1.58 eV) material. However, for the Cs_2_TiI_2_Br_4_ material after 40 °C, V_OC_ starts to drop. On the other hand, Cs_2_TiI_3_Br_3_, Cs_2_TiI_4_Br_2_, Cs_2_TiI_5_Br_1_ and Cs_2_TiI_6_ materials have a lower band gap. So, when the temperature starts to rise, the open circuit voltage (V_OC_) starts to drop after 20 °C onwards despite the existence of almost constant J_SC_. As a result, PCE and P_MAX_ after 20 °C start to reduce with the voltage drop. The same will be evidenced from Equation (2). This means as the temperature starts to rise, V_OC_ starts to decrease continuously and the back recombination process is begun. As a result, the PCE starts to fall gradually. So, from the above analysis, we may conclude that the optimal temperature for the Cs_2_TiBr_6_, Cs_2_TiI_1_Br_5_ and Cs_2_TiI_2_Br_4_ materials is 40 °C and for the Cs_2_TiI_3_Br_3_, Cs_2_TiI_4_Br_2_, Cs_2_TiI_5_Br_1_ and Cs_2_TiI_6_ absorbing layers is 20 °C.

**Figure 5:**
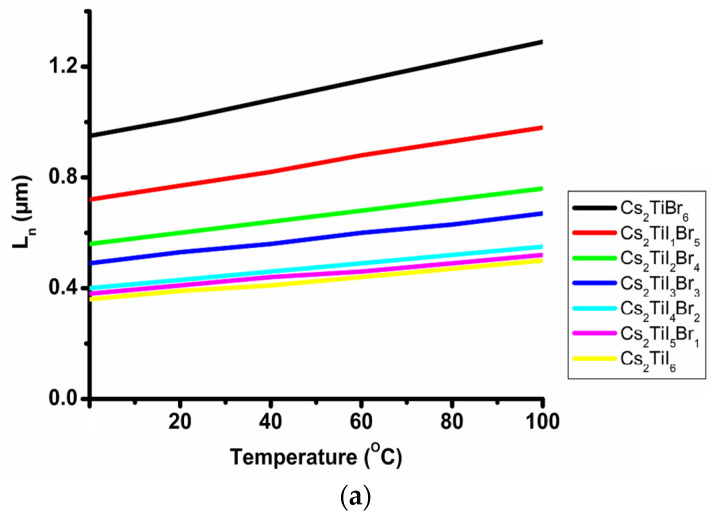

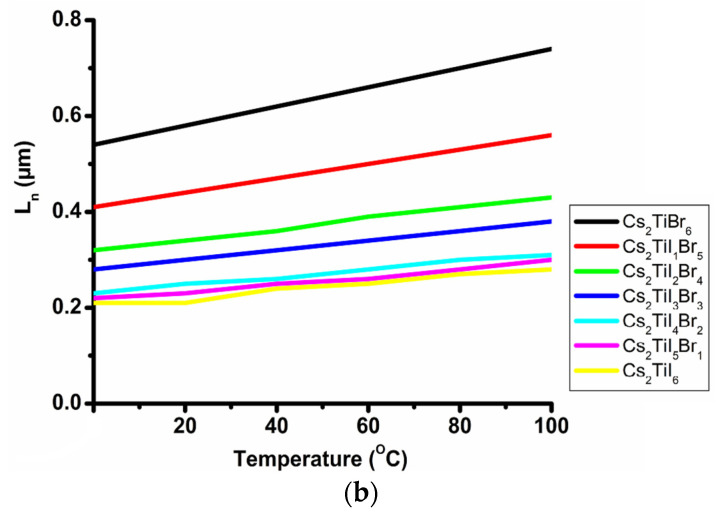
Changes in diffusion length with the working temperature: (**a**) electron, (**b**) hole.

Similarly, changes in electron and hole diffusion length are depicted at 0 °C to 100 °C working temperature in Figure 5a,b. It can be observed that at a relatively higher temperature, the value of L_n_ is increasing simultaneously, as the diffusion length depends on the operating temperature and dopant concentration.

### 3.3. Optimization of Defect Density

**Figure 6:**
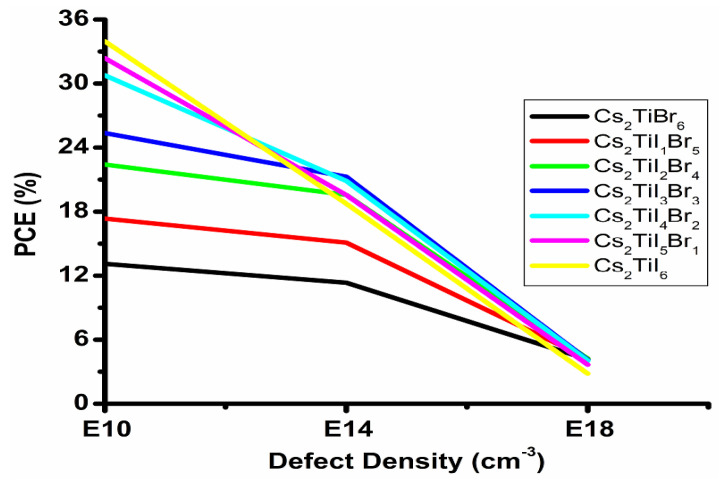
PCE of C–_2_TiI_6−X_Br_X_ absorbing layer with different defect densities at optimized thickness and temperature for CdS ETL.

The total defect density plays a key role in determining the performance of a perovskite solar cell device, as it can debase the performance quality of the device. It has caused heavy charge recombination between the interfaces [25]. In our present study, we have changed the total defect density (N_t_, cm^−3^) from 10^10^ to 10^20^ in the perovskite absorbing layer with a cell architecture of FTO/CdS/Cs_2_Ti–_6−X_Br_X_/CuSCN to understand its effects on the device performances (PCE), and then tried to find out the optimum defect density for all the materials at the optimized thickness and temperature. It is observed that at a higher total defect density, there is a higher recombination of electron and hole pairs due to the generation of pinholes at the electrodes. Such a phenomenon reduces the stability of the device and overall performance of the device. The effects of defect density at the different interfaces are not included in this study. Here, Figure 6 shows the PCE variation with defect density for the Cs_2_Ti–_6−X_Br_X_ absorbing layer with the CdS electron transport layer, respectively. So, from the above figures, it is clearly observed that the increment in defect density above 10^10^ cm^−3^ will reduce the PCE, respectively, for all the perovskite materials. We can also observe that for the Cs_2_TiBr_6_ absorbing material, the PCE decreases significantly with the defect density as it has a higher band gap as a result of the recombination rate starting to reduce. As a result, the collection of electron–hole pairs is also reduced and J_SC_ starts to decrease. Such changes result in the reduction of V_OC_. The Cs_2_TiI_1_Br_5_, Cs_2_TiI_2_Br_4_, Cs_2_TiI_3_Br_3_, Cs_2_TiI_4_Br_2_, Cs_2_TiI_5_Br_1_ and Cs_2_TiI_6_ materials have a continuous V_OC_ drop with J_SC_ reduction, and as a result the PCE falls. This incident signifies that at the higher total defect density, the back recombination process increases due to the impurities’ enhancement in the absorbing materials, and as a result, conversion efficiency (PCE) loss starts to increase due to the V_OC_ and J_SC_ losses [24]. Such losses in the parameters will decrease the P_MAX_ for the absorbing layers which can be observed in Figure 6. So, from the above analysis of the performance parameters, the optimal defect density for the C–_2_TiI_6−X_Br_X_ absorbing layer with CdS ETL is 10^10^ cm^−3^. Hence, it can be concluded that the total defect density of the materials affects the PCE of the device as increasing defects imply reduction in the diffusion length of the electron and hole charge carriers.

## 4. Conclusions

This research article presents the optimization of the thickness of the absorbing layer with two different electron transport layers (CdS) numerically using the SCAPS simulator for the planar FTO/CdS/Cs_2_TiI_6−X_Br_X_ /CuSCN structure. The results indicate the performance of the device (PCE, V_OC_) and maximum power conversion (P_MAX_) are better between 0.5 and 1 µm. Through further observations, we have seen that optimization of the device temperature lies between 10 °C and 60 °C and defect densities between 10^10^ and 10^14^ cm^−3^ for the different absorbing materials. During the simulation process, it was observed that defect densities have a great impact on the charge recombination rate. This research work has not covered the recombination rate in different interfaces of the solar cell device, which include the ETL/perovskite and perovskite/HTL interfaces.

## Figures and Tables

**Table 1:** Device material properties.

Properties	CuSCN	CdS	FTO
Thickness (µm)	0.35	0.50	0.1
E_g_ (eV)	3.40	2.40	3.60
E_a_ (eV)	1.90	4.18	4.0
ℇ_r_	9.0	10.0	9.0
N_D_ (1/cm^3^)	0	1 × 10^15^	2.4 × 10^18^
N_A_ (1/cm^3^)	1 × 10^18^	0	1 × 10^5^
µ_n_ (cm^2^/V_S_)	2 × 10^−4^	100	100
µ_p_ (cm^2^/V_S_)	1 × 10^−2^	25	25

**Table 2:** Basic parameters’ properties.

Properties	Cs_2_TiI_1_Br_5_	Cs_2_TiI_2_Br_4_	Cs_2_TiI_3_Br_3_	Cs_2_TiI_4_Br_2_	Cs_2_TiI_5_Br_1_	Cs_2_TiBr_6_	Cs_2_TiI_6_
Thickness (µm)	0.3–4	0.3–4	0.3–4	0.3–4	0.3–4	0.3–4	0.3–4
Band gap, E_g_ (eV)	1.58	1.38	1.26	1.15	1.07	1.78	1.65
Electron affinity, E_a_ (eV)	3.42	3.62	3.74	3.85	3.93	4.47	4.20
Relative permittivity, ℇ_r_	10	19	22	25	28	10	18
Donor density, N_D_ (1/cm^3^)	1 × 10^19^	2 × 10^19^	1 × 10^18^	5 × 10^18^	5 × 10^18^	1 × 10^19^	9 × 10^18^
Acceptor density, N_A_ (1/cm^3^)	1 × 10^19^	2 × 10^19^	1 × 10^18^	5 × 10^18^	5 × 10^18^	1 × 10^19^	9 × 10^18^
Electron mobility, µ_n_ (cm^2^/V_S_)	4.4	5.4	5.8	7.8	7.8	4.4	8.4
Hole mobility, µ_p_ (cm^2^/V_S_)	2.5	2.9	3.1	3.9	3.9	2.5	4.3

## Data Availability

The data presented in this research are available on request from the corresponding author.
